# The feasibility of community mobilisation for child injury prevention in rural Nepal: a programme for female community health volunteers

**DOI:** 10.1186/s12889-015-1783-5

**Published:** 2015-04-28

**Authors:** Puspa Raj Pant, Bharat Budhathoki, Matthew Ellis, Dharma Manandhar, Toity Deave, Julie Mytton

**Affiliations:** Centre for Child and Adolescent Health, University of the West of England, Bristol, UK; Mother and Infant Research Activities (MIRA), Kathmandu, Nepal; School of Social and Community Medicine, University of Bristol, Bristol, UK

**Keywords:** Community mobilisation, Women’s group, Child injury, Educational intervention, Injury prevention, Nepal

## Abstract

**Background:**

Injuries accounted for 23% of all deaths in children and adolescents in Nepal during 2010 (n = 3,700). Despite this, there is no national death registration or injury surveillance system. Non-fatal injuries are many times more common than fatal injuries and may leave the injured person with lifelong consequences. Children in low-income settings are exposed to widespread risks of injuries but there is little awareness of how they can be prevented. Community mobilisation has been shown to be effective to reduce maternal and neonatal morbidity. This study aimed to develop a child safety programme and assess the feasibility of delivering the programme through a community mobilisation approach.

**Methods:**

We developed a culturally appropriate, educational programme for Female Community Health Volunteers that included both primary and secondary prevention materials for unintentional child injuries. We determined the feasibility of evaluating its effectiveness through the mobilisation of women’s groups in rural Nepal. Ten women’s groups across 9 wards in one village development committee area completed the programme during 6 monthly meetings. Parent-reported injuries were collected through a notification system established for this study. Experience of the programme by women’s group participants and leaders was assessed through a structured questionnaire and process measures assessed the delivery and reach of the programme.

**Results:**

Programme resources were developed for this setting and adapted following feedback from users. Nine FCHVs received first-aid training and shown how to use the facilitation manual and injury prevention resources. The FCHVs convened 10 women’s groups to run over 6 months with 24–29 mothers attending each meeting (290 mothers participated in total). Each group presented their views on child injury risks and proposed prevention activities at local public meetings. Women reported 155 injuries to children under 18 years during 7 months of follow up using the notification system.

**Conclusions:**

It is feasible to develop and implement a community mobilisation intervention where women’s groups work together with local FCHVs to prevent injuries in children. The intervention was well received by the women’s groups and by community members. The effectiveness and cost effectiveness of the intervention should now be evaluated through an experimental study.

**Electronic supplementary material:**

The online version of this article (doi:10.1186/s12889-015-1783-5) contains supplementary material, which is available to authorized users.

## Background

Child injury is a major global public health problem with its highest burden in low and middle income countries [1]. In Nepal, injury was estimated to be the cause of 3,723 deaths among children and adolescents aged 1–19 years in 2010 [2]; in the absence of national death registration and injury surveillance systems, this is likely to be an underestimate of the true amount [3]. As a proportion of all child deaths (1–19 years) in Nepal, deaths due to injury have risen from 13% in 1990 to 23% in 2010. Fatal injuries are much less frequent than non-fatal injuries [1,4,5]. Non-fatal injuries may leave life changing consequences contributing many kinds of disabilities [1,6,7].

There are few systematic data for describing the epidemiology of non-fatal injuries in Nepal. There are only a few population-based studies on child injuries conducted in South-East Asian countries [8]. Government reports of hospital admissions classified using ICD10 codes, show that approximately 7,000 children and adolescents (aged 0–19 years) are hospitalised due to injuries annually [9-12]. Fractures, burns/corrosions, poisoning, road traffic injuries and falls account for half of these hospitalisations. A study of emergency department attendances at 11 tertiary hospitals of Nepal recorded 38,000 injuries during 2008/09; about 30% of these cases were children below 15 years [13]. These reports miss the cases taken to bordering Indian hospitals and those treated in local private clinics. Due to the distance from healthcare facilities and the cost associated with seeking healthcare, many child injuries do not present to healthcare settings. This implies that the actual number of injury-related hospitalisations may be much higher. A Global Road Safety Report estimated that 1,000 deaths, 18,000 hospitalisations and 143,000 injuries not requiring hospitalisation of children and adolescents in Nepal in 2010 were due to road injuries alone [14]. Using these estimates, there would be 161 non-fatal road injuries for every child dying on the road.

Child labour is common in Nepal and may expose children to specific additional hazards. Forty percent of all children aged 5 to 17 years in Nepal are classified as ‘working children’ [15]. Almost 70% of the children in Nepal are deprived of at least one of the seven indicators mentioned in the Bristol deprivation score calculation [16] i.e. food, shelter, sanitation, water, information, education and health. Forty percent of Nepali children are thought to be deprived of at least two of these indicators [17]. Almost half of Nepal’s population is comprised of children below the age of 18 years [18]. About 80% of the country’s population lives in rural areas but the rate of urbanisation is 5% per year i.e. increase in urban population [19]; 39% of its population living below the poverty lines of 1.25 US Dollar a day [20]. Nepal thus has many factors that are likely to contribute to its increased risk of injuries.

There are few published trials of child injury prevention interventions in low-income settings. Social mobilisation for child injury prevention is recommended in low-income settings as it has been shown to be effective [21,22]. In Bangladesh, community-based interventions were found to reduce the rates of injury related hospitalisation by one-third [22]. Community mobilisation has been proven to be effective and cost-effective to improve maternal, child and perinatal health in rural Nepal; the mobilisation of local women’s groups led by female community health volunteers (FCHVs) has been well documented [23-25]. A community survey of child injuries and qualitative study exploring injury prevention awareness has been conducted in this setting [26,27]. We describe the development of a participatory intervention utilising women’s group mobilisation for the prevention of child injuries and a study exploring the feasibility of delivering and evaluating such an intervention in rural Nepal.

## Methods

### Setting

**Figure 1:**
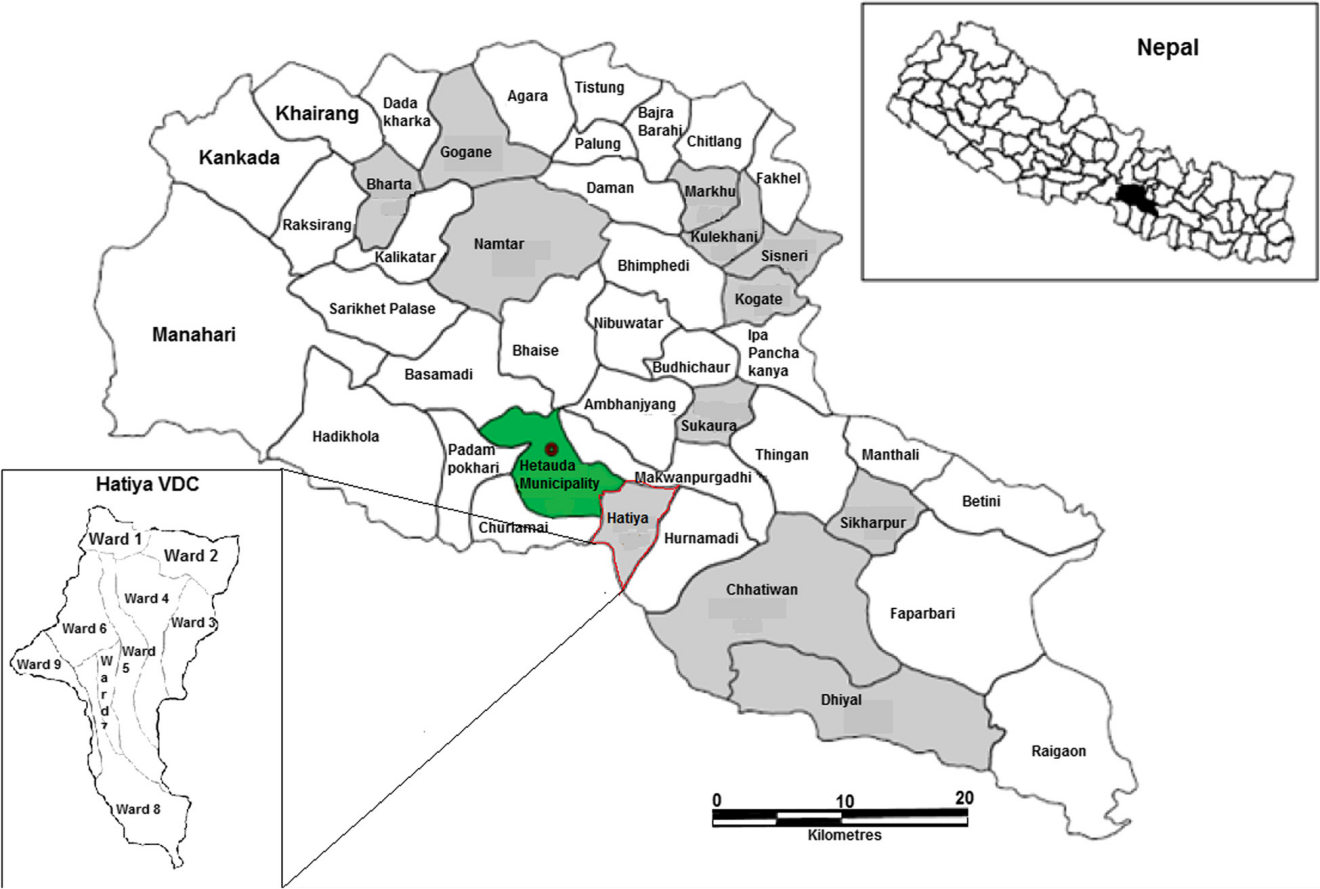
Map of Makwanpur district, showing Hatiya VDC and wards.

The project was based in Hatiya Village Development Committee (VDC) in Makwanpur district and was conducted between September 2013 and May 2014. Makwanpur district represents typical rural Nepali settings and is described in detail elsewhere [23,27]. There is one main urban centre, Hetauda. Hatiya VDC has a population of 13,000 in 2,750 households across 9 wards [28]. Most of the families are subsistence farmers often supplemented by remittances sent by one of the family members working abroad. People also work in a local cement factory. There is one government health-post which provides basic care such as immunisations, ante-natal and post-natal services. A paramedic (Health Assistant) is incharge of the health-post and nine Female Community Health Volunteers (FCHVs), who are local women, are responsible for providing preventive mother and child health care and education in the community. In Hatiya, each FCHV covers an area of between 400–900 households. The FCHV usually delivers health promotion through facilitated discussions at monthly women’s group meetings held in each ward. In general, a women’s group has at least 20 members, including mothers of children of any age (0–17 yrs). In ward number 5, there are two women’s groups due to the elongated shape of the ward (Figure 1).

Women’s groups usually convene on a fixed day every month and are well established, having run for over a decade in Hatiya VDC. Usually the FCHV will bring health-related issues to the group to discuss; a micro saving & credit activity also forms part of the routine agenda of the meeting. Because the groups have been operating for such a long time, the age of the members ranged from twenties to fifties. Details of the wards and women’s groups in Hatiya VDC are shown in Table 1.

**Table 1:** **Distribution of population of Hatiya VDC in 2011 by wards**

**Ward no**	**Households**	**Total population**	**Female**	**Children (<18 yrs)**	**Mothers’ groups**	**Average attendance**
1	183	863	444	388	1	25
2	179	1,003	497	451	1	27
3	434	1,941	1,016	873	1	24
4	368	1,769	895	796	1	29
5*	339	1,638	520	737	2	25 and 26
6	278	1,284	676	579	1	27
7	221	1,058	546	476	1	27
8	392	1,858	966	836	1	25
9	357	1,685	860	758	1	25

*ward number 5 stretched north–south and therefore has two women’s groups in both ends, covered by one FCHV.

### Intervention development

The intervention was adapted from two existing community-based programmes. The injury prevention component was adapted from the PRECISE (**PRE**vention of **C**hild **I**njuries through **S**ocial intervention and **E**ducation) study in Bangladesh [22] which developed and evaluated injury prevention packages for home, school and community settings. The programme was developed to cover a number of common unintentional injuries in children including falls, drowning, burns, poisoning, animal and road traffic injuries. Materials were developed and adapted following feedback from FCHVs and the women in the groups immediately after each session. They included a facilitation manual for the FCHVs to use in the women’s group meetings, picture books and posters using images by a local artist, and the resources for collecting feedback on the programme and parent-reported injuries in the children.

The delivery and evaluation component of the study was developed with Mother and Infant Research Activities (MIRA); a non-governmental organisation who have been working in Makwanpur for 14 years and are the national leader in the development of women’s groups for health [23]. The six injury prevention sessions (Table 2) were designed through adaption of the 10 sessions employed for the MIRA trial described in Manandhar et al. [23] and Morrison et al. [24].

**Table 2:** **Summary of the contents and techniques for women’s group meeting sessions**

**Meeting**	**Agenda/Contents of the meeting**	**Methods**
1	• Brief information on the objectives of MIRA projects	Discussion, game, pictures
	• Objectives of the Child Injury Prevention project	
	• UN CRC definition of a child/who is a child?	
	• Definition of an injury and its classification	
	• Common injuries to children and places of occurrence	
	• Exchange of information about the types of child injuries occurring in the neighbourhood: non-fatal, disabling or fatal	
	• Discussion of child supervision systems in families and community	
	• Concept of the community about child injuries	
	• Discussion on the practice of treatment of injured children	
2	• Brief Review of last month’s meeting	Discussion, story, picture, game, interaction
	• What have you (women’s group members) done in the past month? How was the injury data collection work?	
	• Make a list of the problems faced by a family when a child is injured, disabled or died due to injury	
	• Discussion on – what is wise? doing medical treatment after an injury or preventing it? which has more benefits? why and how?	
	• Discussion on the importance of first-aid to an injured person	
3	• Brief review of last month’s meeting	Discussion, picture, interaction
	• What have you (women’s group members) done in the past month? How was the injury data collection work?	
	• Discussion about the measures applied by your own household and the community for injury prevention	
	• What are the challenges and barriers of child injury prevention in our community?	
	• Group discussion about identifying all risks and hazards for child injuries and measures to remove them.	
	• Identify the ways to prevent many children collectively from injuries	
4	• Brief review of last month’s meeting	Discussion and development of a plan
	• What have you (women’s group members) done in the past month? How was the injury data collection work?	
	• Preparation for a ward level mass meeting to inform the community about child injury prevention activities done in the last three meetings	
	• Ensure the presence of child club members in the ward level mass meetings	
5a	*This meeting should be called one week before ward level meeting:*	*Meeting with nominated members only*
	• Ensure the invitation for the meeting has reached to all invitees	
	• Prepare the presentation for the meeting by the members nominated in previous meeting	
5b	• Disseminate the activities of the women’s group to the community	Group discussion and interaction
	• Seek support and advice from the ward level leaders	
	• Discuss the identified challenges and make a plan to overcome them in consensus	
6	• What have you (women’s group members) done in the past month? How was the injury data collection work?	Discussion and interaction
	• Conduct a review analysis of the ward level mass meeting	
	• Make rules and strategy to implement to plans presented at the ward level meeting.	
	• Make a VDC level coordination committee to overcome any problems that may arise in the future.	
	• Prepare for a VDC level meeting	

This study defined a child as a person who is aged below 18 years. Ethical approval was obtained from Nepal Health Research Council (NHRC) and the research ethics committee of the University of the West of England, Bristol, UK. Verbal consent was obtained in group from FCHVs and women’s group members in every wards of the project VDC whereas parental consent was obtained before the data collection for injured children.

### Feasibility testing of the intervention

The feasibility of delivering the intervention was tested over a total of six months across all 10 women’s groups in Hatiya VDC. To engage FCHVs and to raise injury awareness, all nine FCHVs were offered a four day first-aid course run by the Nepal Red Cross and were provided with a first-aid kit. FCHVs were asked to support the field testing by encouraging attendance at the women’s group meetings and to monitor the number of women who attended each session. Training was provided for the FCHVs on child injuries and how they can be prevented and in how to use the facilitation manual, the posters and the picture book. The resources were planned to be used in six consecutive women’s group meetings which each lasted 1–2 hours. Feedback from FCHVs, chairs of the women’s groups and local leaders was collected at the end of the six months using a structured questionnaire that asked questions about its feasibility and acceptability.

We also tested the feasibility of using parent-reported injury outcomes within the study. An injury identification and notification system was developed encouraging mothers to report any child injury that had occurred during the past month. An injury case was defined as an unintentional injury that required treatment, or resulted in missing school or work or being unable to carry out activities of daily living for at least one day. Injury caused by sharp objects, drowning, poisoning, falls, injuries caused by animals, electric shocks, burn/fire, suffocation, injury caused by falling objects, injury caused by machines and transport related injuries were included. A two-page injury reporting form (mostly comprised of illustrative pictures) was developed in Nepali language and distributed to FCHVs and mothers’ group members. The questionnaire was discussed in each mothers’ groups during the meetings and ensured that they understand the concept and are able to complete the form. The VDC facilitator (a local female recruited by the project to supervise the FCHVs and group activities) supported the monthly completion and collection of the forms. This activity was also related to the mothers’ group meeting discussion agenda; with an objective to know how many children in their community suffer injuries.

### Outcome measures

**Figure 2:**
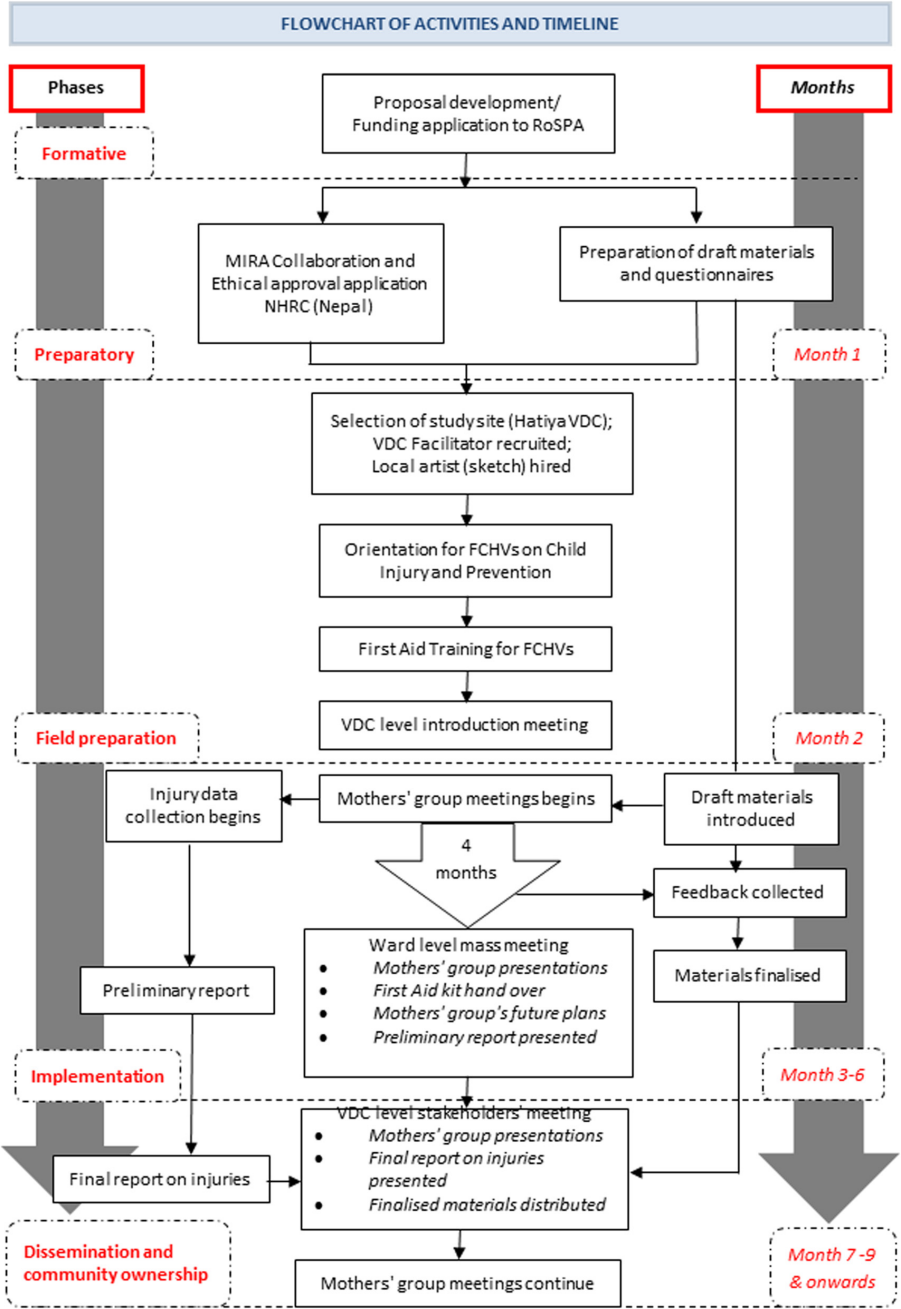
Flowchart of the project activities.

The feasibility of the intervention was evaluated by, 1) the number of women’s group meetings where child injury and the facilitation resources were used, 2) attendance of the group members in meetings, 3) acceptance of the intervention by FCHVs, 4) acceptance of the intervention by women’s group members, 5) acceptance of the intervention by community people, 6) child injury prevention initiatives identified by the women’s groups and proposed at the ward level meeting at the end of the intervention period. The feasibility of testing its impact using community-based injury reporting was assessed with reference to the number of injuries reported by parents during the six months of intervention delivery (Figure 2).

## Results

### Development of intervention resources

Thirteen resources were prepared during the intervention development phase and tested and finalised in the Nepali language. The materials and tools prepared by this project were:

1. Women’s group Meeting Facilitation Manual
  Picture Book *(Supporting materials for meeting 1-6)*
  Posters *(Supporting materials for meeting 1-3)*
  Milestones for child development: from birth to 11 years *(Supporting material for meeting 1)*
  Stories related to child injuries *(Supporting material for meeting 2)*
  Checklist for making home safe from injuries *(Supporting material for meeting 3)*
  Ward meeting schedule *(Supporting material for meeting 5)*
  VDC meeting schedule *(Supporting material for meeting 6)*
2. FCHV child injury knowledge assessment used before and after orientation and training session
3. Attendance monitoring form for FCHVs *(Monitoring form)*
4. Observation of the meeting and feedback form for the VDC facilitator *(Monitoring form)*
5. Feedback form for FCHVs *(Monitoring form)*
6. Child injury data collection form (Additional file 1).

### Delivery of the intervention

Implementation of the intervention in Hatiya VDC was facilitated effectively by MIRA and gained cooperation of the community. All the nine FCHVs accepted and actively participated in the four-day first-aid training. The nine FCHVs convened 10 women’s group (one in each ward except two in ward number 5) each month for six months. Average attendance in the group sessions ranged between 24–29 women (Table 1).

In contrast to the findings of a previous study in the same VDC area [27], women participating in the intervention groups believed that the injuries experienced by their children were often preventable. They recognised that they were distressed to see their own child living in pain after an injury event. The attitude of women’s group members was considerably changed after taking part in the first three consecutive meetings, where various aspects of child injuries, the importance of preventing them and brainstorming for possible solutions took place.

The women attending all 10 groups were able to demonstrate they had started to identify the risks for child injuries in their communities and identify options to minimise those risks. During ward level public meetings, where local leaders and social workers were present, they conveyed the message that the proposed interventions were for their own children, their growth and development, more importantly saving life and money. There was also an opportunity to get support and coordination from other community groups active in the village. Strategies and actions to reduce injury risks reported at these meetings included: identification of risks and hazards, involving community groups in implementing activities such as – filling up the ditches, putting a fence around the roof and balcony of houses, increasing child supervision and continuing to discuss child injury prevention in further women’s group meetings.

The local leaders and social workers were universally supportive of the suggestions made by the women’s groups. For example, the adolescent girls’ club showed their interest in supporting the women’s group activities, particularly in helping the reporting of children who had sustained injuries and in spreading the prevention messages.

In addition to increasing awareness about child injury prevention among the mothers, an unanticipated observation was that this programme helped raising their self-confidence. During the ward level meeting, many mothers were speaking in public for the first time. After this meeting they reported that they felt able to speak in public on behalf of their children. This has, reportedly, increased their enthusiasm. During the ward level meeting, all of the women’s groups announced that the women’s group had established a Child Injury Prevention Fund and requested support from the ward and VDC to match the contribution. Starting an Injury Prevention Fund was also a sustainable idea of the women’s groups where donations were made in each ward to cover the costs of replenishing the first-aid box and providing loans towards the cost of emergency treatment of child injuries.

### Acceptability of the intervention

Feedback on the acceptability of the intervention was collected at the end of the monthly meetings using structured tools. Questions in the feedback forms related to structure and timing of the sessions. It was observed that some participants were not confident in providing feedback, having not previously been asked to input to the development of resources in this way. Support and encouragement helped the FCHVs and women’s group leads to complete the feedback forms with the help of the VDC facilitator. Feedback that led to direct changes to the resources included the addition of examples of child injuries and their consequences, an increase in the number of pictures and illustrations, and an increase the font size.

After all six sessions had been delivered; feedback was collected from 30 community members, including FCHVs, women’s group chairs, and community leaders/social workers. In order to quantify the perception of different group of community people, a set of questions were developed where the responses were measured on a scale from 1 to 5 (Tables 3, 4 and 5).

**Table 3:** **Opinions of the FCHVs on acceptability of the intervention (N = 9)**

***Q. No. Statements***	***Strongly disagree (1)***	***Disagree (2)***	***Can’t say (3)***	***Agree (4)***	***Strongly agree (5)***
1. Child injury prevention is an important issue to consider for children’s health and development	-	-	-	9 (100%)	-
2. Before this programme, I knew that injuries to children are preventable	-	-	2 (22.2%)	7 (77.8%)	-
3. I think there is a need for such an intervention in the community	-	-	-	5 (55.6%)	4 (44.4%)
4. The intervention helped me learn about child injury prevention	-	-	-	6 (66.7%)	3 (33.3%)
5. I think other parents/carers will also find this intervention useful	-	-	1 (11.1%)	8 (88.9%)	-
6. The total duration of the intervention i.e. monthly sessions for 6 months is appropriate	-	4 (44.4%)	-	5 (55.6%)	-
7. The duration is too long	-	-	-	-	-
8. The duration is too short	-	-	-	4 (44.4%)	-
9. The duration of the sessions for about 2 hours is appropriate	-	1 (11.1%)	-	8 (88.9%)	-
10. The duration of sessions is too short	-	-	-	-	-
11. The duration of the sessions is too long	-	-	-	1 (11.1%)	-
12. The duration between the sessions i.e. one month is appropriate	-	-	-	9 (100%)	-
13. The monthly interval is too long	-	-	-	-	-
14. The monthly interval is too short	-	-	-	-	-
15. The session facilitation manual appropriately covers the issues related to child injury prevention	-	-	-	8 (88.9%)	1 (11.1%)
16. Layout and the design of the manual are appropriate	-	-	-	8 (88.9%)	1 (11.1%)
17. Participation of the Mothers in the meeting is appropriate	-	-	-	9 (100%)	-
18. Women’s group can lead to organise more sessions of child injury prevention themselves	-	-	1 (11.1%)	8 (88.9%)	-
19. I will be able to communicate these concepts with parents I see	-	-	-	4 (44.4%)	5 (55.6%)
20. The intervention is relevant to my everyday work	-	-	-	8 (88.9%)	1 (11.1%)

**Table 4:** **Opinions of local social workers on acceptability of the intervention (N = 11)**

***Q. No. Statements***	***Strongly disagree (1)***	***Disagree (2)***	***Can’t say (3)***	***Agree (4)***	***Strongly agree (5)***
1. Child injury prevention is an important issue to consider for children’s health and development.	-	-	-	10 (90.9%)	1 (9.1%)
2. I think people ignore injuries because they don’t think it can be prevented	-	4 (36.4%)	3 (27.3%)	4 (36.4%)	-
3. Before this programme, I knew that we can prevent children from injuries	-	-	1 (9.1%)	8 (72.7%)	2 (18.2%)
4. I think there is a need for such an intervention in the community	-	-	-	7 (63.6%)	4 (36.4%)
5. I think other parents/carers will also find this intervention useful	-	-	2 (18.2%)	8 (72.7%)	-
6. I think women’s group meetings with FCHVs are useful to prevent child injuries in the community	-	-	-	9 (81.8%)	2 (18.2%)
7. Women’s group can lead to organise child injury prevention activities after this programme	-	-	2 (18.2%)	9 (81.8%)	-
8. I have observed the meetings and the educational materials are appropriate	-	-	3 (27.3%)	8 (72.7%)	-
9. I will be able to communicate these concepts with parents I see	-	-	-	10 (90.1%)	1 (9.1%)
10. The intervention is relevant to my everyday work	-	-	1 (9.1%)	10 (90.9%)	-

**Table 5:** **Opinions of the women’s group chairs on acceptability of the intervention (N = 10)**

***Q. No. Statements***	***Strongly disagree (1)***	***Disagree (2)***	***Can’t say (3)***	***Agree (4)***	***Strongly agree (5)***
1. Child injury prevention is an important issue to consider for children’s health and development	-	-	-	10 (100%)	-
2. Before this programme, I knew that injuries to children are preventable	-	-	1 (10%)	9 (90%)	-
3. I think there is a need for such an intervention in the community	-	-	-	4 (40%)	6 (60%)
4. The intervention helped me learn about child injury prevention	-	-	-	8 (80%)	2 (20%)
5. I think other parents/carers will also find this intervention useful	-	-	-	10 (100%)	-
6. The total duration of the intervention i.e. monthly sessions for 6 months is appropriate	-	3 (30%)	-	6 (60%)	1 (10)
7. The duration is too long	-	-	-	-	-
8. The duration is too short	-	-	-	3 (30%)	-
9. The duration of the sessions for about 2 hours is appropriate	-	-	-	10 (100%)	-
10. The duration of sessions is too short	-	-	-	-	-
11. The duration of the sessions is too long	-	-	-	-	-
12. The duration between the sessions i.e. one month is appropriate	-	-	-	10 (100%)	-
13. The monthly interval is too long	-	-	-	-	-
14. The monthly interval is too short	-	-	-	-	-
15. I think such meetings with FCHVs are useful to prevent child injuries in the community	-	-	-	9 (90%)	1 (10%)
16. I will be able to do the activities suggested by FCHVs/this Programme for the safety of my child	-	-	-	8 (80%)	2 (20%)
17. Women’s group can lead to organise more sessions of child injury prevention themselves	-	-	-	9 (90%)	1 (10%)
18. I don’t think I will have enough time to attend the meeting**	-	-	2 (20%)	4 (40%)	4 (40%)
19. My family will support such an intervention	-	-	-	10 (100%)	-
20. I will be able to communicate these concepts with parents I see	-	-	-	8 (80%)	2 (20%)

**Note: traditionally, rural females are laden with daily routine of fetching wood and water, working in the field and cattle, feed the children and family, washing and cleaning which barely give them time to think about personal development. This leads them to believe that they might not have time to attend a meeting. Yet these women’s group members managed to attend monthly meetings regularly.

Key findings emerging from this feedback included strong support for a programme addressing child injury prevention from FCHVs, community leaders/ social workers and from women’s group chairs. There were varying opinions about whether the intervention was the right number of sessions, with a small proportion believing that the intervention was too short (four FCHVs (44%) and three women’s group chairs (30%)). To enable them to attend the meetings, all women’s group chairs said that they had received the support of other family members. However, many (80%) of them were concerned that they have limited time to attend these meetings as they have many other domestic tasks to carry out. It was also observed that they adjusted the times of the meetings to early morning or during the mid-day break to allow the groups to continue, despite them occurring during the period when there was a lot of labour required for the cultivation, harvesting or processing of crops.

### Parent-reported child injury data collection

A total of 155 children were injured during the 7 months; 65% of these injuries occurred at home. Falls were the major cause of injuries and accounted for 61% of all injuries to children aged <18 years. The non-fatal injury rate for the entire VDC was calculated to be 45 per 1,000 children which ranged across the wards from as low as 26/1,000 to a high of 88/1,000. These rates are 25% higher than rates in an earlier study [26]. The proportion of home injuries and the gender ratio were similar in both studies, however, the higher injury rate might also have resulted from a modification in the definition and from the mobilisation of community members to undertake the data collection.

About half of the injured children (75/155) received treatment. Information provided by the mothers in the groups indicated that average treatment costs per injury were 4,900 Rupees or 50 US$ (15% of per capita average annual income of Nepal). Families have to pay the costs of treatment themselves, and these costs placed a considerable burden on some families. It was observed that the women were able to administer the data collection tool (Additional file 1) following a brief orientation session. It was also observed that, for the injuries with no visible signs or those with short recovery time, to collect the necessary information, some probing was needed, therefore they may still have underestimated the true rate of injuries occurring.

## Discussion

This paper describes the development and feasibility testing of a community mobilisation intervention to prevent child injuries in rural Nepal; educational intervention was an important aspect of it. We have shown that it is feasible to engage FCHVs and women’s groups in the programme, and to collect parent-reported child injury outcomes through a community supported data collection system.

### Strengths

To our knowledge, this study is the first of its kind conducted in Nepal. The major strength of this study is that it included both primary (safety information) and secondary (first-aid training) prevention components and developed resources using community groups in the setting in which it is intended to be used. The intervention builds upon evidence that women’s group mobilisation for health promotion has been proven to be effective and cost-effective in rural Nepal [23-25,29]. At the outset, the intervention promoted community participation and ownership. The first meeting was very important as it set out the context. Interactively, participants provided details of child injury events they had witnessed, treatment costs and other practical issues related to the care of injured children in subsequent sessions. Discussions enabled them to conclude that prevention of children from injuries is important and that it is the major responsibility of parents and carer. In the beginning, the mothers referred to severe child injuries in the past and compared them with disability or not being able to attend school.

The injury data collection undertaken concurrently with women’s group meetings showed that it is feasible to collect information at a community level. When these results were fed back to the women’s groups, they conveyed a clear message that child injury was an issue for their community. They were able to count how many children suffered injuries in their neighbourhood and also knew how much money had been spent on their treatment. Presenting these figures in public had a considerable positive impact on community involvement, cooperation and future collaboration among local stakeholders.

The concept of injury prevention has not been recognised in Nepali society but is very much needed in the country today. In a previous qualitative study, community members expressed their unfamiliarity with this issue and were keen to be involved in future endeavours [27]. Their genuine interest to be involved was reflected in their participation in this project. This project started its field activities just before the two Nepali festivals of Dashain and Tihar (in October) which also coincide with the paddy and maize harvesting season. However, mothers arranged their meeting early in the morning in order not to miss participation, thus maximising attendance despite the need to collect the harvest. This demonstrated their commitment to the issue. The FCHVs also showed their commitment by completing the four-day long first-aid training just before the festival holidays; one of the FCHVs had her son’s wedding during the first-aid training but she didn’t take time off from the training.

### Sustainability of the activities

The importance of sustainability for the success of a programme is well recognised [22-25,29]. We have attempted to develop a sustainable intervention through the engagement of the community during the development and testing of the materials and the development of simple resources that can be produced relatively cheaply and re-used. To assure the sustainability of this project we planned the following: 1) first-aid training, 2) facilitation manual for use in the meetings, 3) educational materials, and 4) a child injury prevention fund. The first-aid training undertaken by the FCHVs boosted their self-confidence to manage injuries that occurred and resulted in recognition of the FCHV’s role by the community. The activities carried out during this intervention helped to develop the capacity of the women’s group members, particularly in the identification of child injury and speaking in public; this has also been demonstrated by earlier studies [30]. Similarly, the women’s groups received the first-aid kits for the first time which, since established, has augmented their interaction with FCHVs for its use and replenishment. Above all, this intervention assisted women’s groups in exploring solutions, using local resources, and did not promote any one particular activity. Therefore, the community owned the injury prevention strategies and identified the required resources consequentially. Because it requires minimal intervention from external agents, the sustainability can easily be assured. All the women’s groups presented their future plans for the implementation of them in their communities. Many of their activities were about keeping this discussion alive in the mothers’ groups. In addition, they had plans to identify local stakeholders in local authorities to ask for their cooperation and support. People’s responses were found to be very much in favour of the programme. From the findings, we can understand that there are still areas of improvement but that the intervention is generally well accepted by the community.

### Limitations

This study aimed to develop the intervention and explore the feasibility of using the materials in the setting for which they were designed. It does not tell us about the efficiency and effectiveness of the intervention. During the planning phase it was assumed that all the FCHVs and women’s groups would be proficient in conducting group meetings in a well-organised manner because these groups had existed for years. It was observed that some FCHVs were not confident in facilitating groups and some were not fully literate. Therefore, inclusion of brief training on organisational and facilitation skills would be helpful to enable better utilisation of available resources and time.

Studies that have proven the effectiveness of women’s group mobilisation for improving maternal and neonatal health outcomes used a 12 month action learning cycle in which a six month intervention period was followed by a further six months of follow up in which learning can become embedded and behaviour change initiated. The funding available for this study only covered the six months of intervention delivery, and therefore longer term outcomes are not known. We have only tested the intervention in one VDC and in 10 women’s groups, therefore we do not know whether different VDCs and different districts would be able to implement the intervention as well as in Hatiya. We had the benefit of working with MIRA who had existing good relationships with the communities and it is not known how important this was in determining the success of the feasibility study.

A relatively low level of literacy was anticipated to be one of the barriers to engagement; however the participants proved their proficiency differently. The older FCHVs asked their school-going grandchildren to read out the first-aid manual for them. These FCHVs reported that their grandchildren also found the first-aid manual important. Some people expressed their concern about the workload of the FCHVs but these volunteers were seen to possess excellent skills in dividing tasks. FCHVs led and delivered the sessions up to the level that their level of literacy allowed. However, they were also supported by members of the women’s group to read out the excerpt from the manuals and to conduct the discussions. Inclusion of a greater number of pictures in the facilitation manual to illustrate different situations and risks improved the confidence of the FCHVs to deliver the content.

The injury reporting form did not have an identifier for the person completing the form (Additional file 1) due to which it is not possible to state the exact proportion of the mothers’ group members completing the forms. Despite of the parent-reported child injury data collection system covered all households in the wards, an underestimation was revealed during the local dissemination of the figures. The data collection support could have utilised other existing community groups (child clubs, adolescent girls’ groups) with brief orientation on completing the forms in order to achieve higher recall and response.

## Conclusions

This project successfully developed a child injury prevention intervention for rural settings in Nepal. The information generated by this study will be useful for developing a trial to assess whether this programme can prevent injuries among children and whether that intervention is cost effective. The issues identified during the feasibility study and the feedback gained from participants has been incorporated into later versions of the resources and will inform the design of a future trial.

## Additional file

Additional file 1:
**Child injury data collection form.**
