# Supplementary material for: The feasibility of community mobilisation for child injury prevention in rural Nepal: a programme for female community health volunteers
Source: BMC Public Health. 2015 Apr 28;15:430. doi: 10.1186/s12889-015-1783-5 (PMC4418066; doi:10.1186/s12889-015-1783-5)
Supplement: Additional file 1: — Child injury data collection form. [file 12889_2015_1783_MOESM1_ESM.pdf]

Child injury data collection form

VDC: Hatiya, Locality [ ] Ward [ ] Date [ ][ ][ ][ ] [ ][ ] [ ][ ]

Child's name [ ] Age [ ][ ] Year [ ][ ] Month Gender [ ] [ ]

Education [ ][ ] Completed Number of siblings [ ][ ]

Age of mother [ ][ ] Years Education [ ][ ] Occupation [ ] Total children [ ][ ]

1. Roofing material of this household? (please tick one)

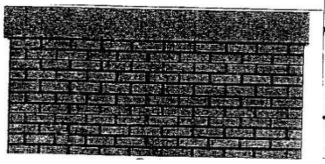
Cement bonded

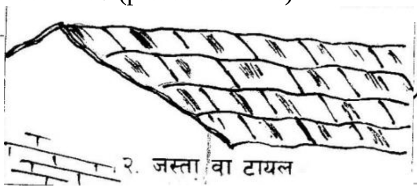

Baked tiles or zinc plate

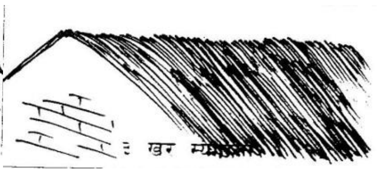
Thatch

2. Place of injury? (please tick one)

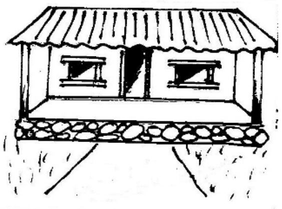 Home	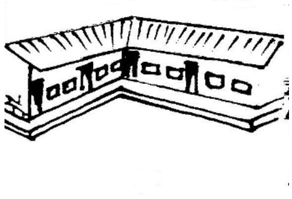 School	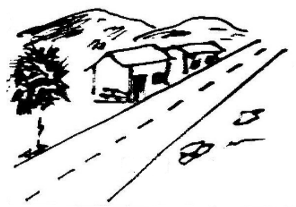 Road/street	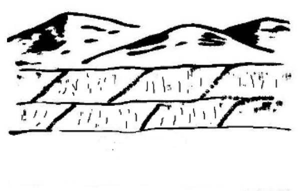 Farm/Fields
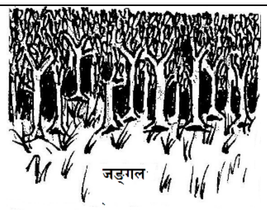 Jungle	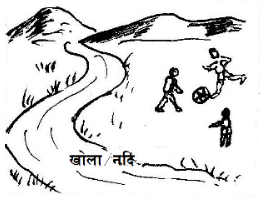 River/streams	Others	

3. Activity of the child at time of injury? (please tick one)

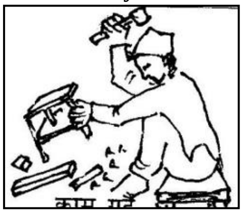
Working

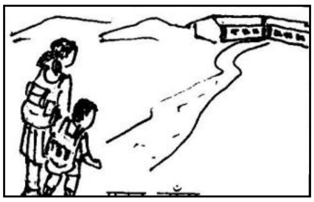
Going school

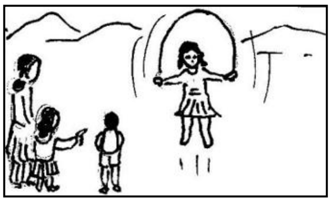
Playing

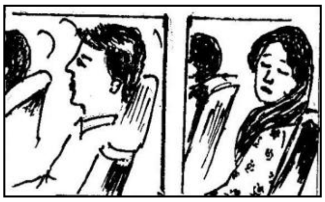
Travelling

Other specify ____

4. Body part injured by the injury [ ]

Did the injury affect their usual activities? [ ] Yes [ ] No

Did the injury cause disability to the child? [ ] Permanent disability [ ] Temporary disability [ ] None

If yes please state [ ]

5. What was the mechanism of injury to child? (Please tick one)

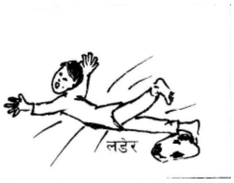

Falling

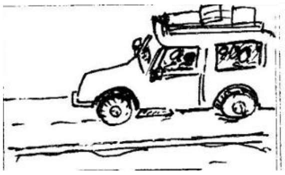

Road injury

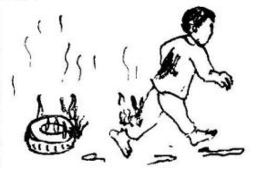

Fire burn

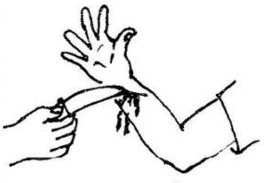

Cut/wound

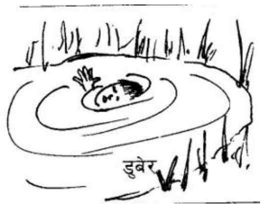

Drowning

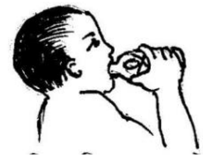

Poisoning

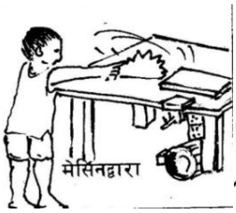

Mechanical

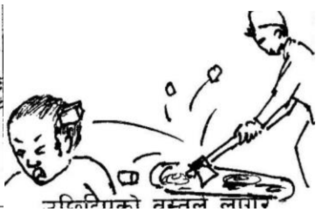

Hit by moving object

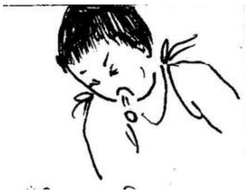

Choking

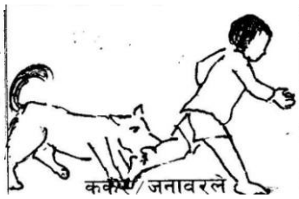

Animal/dog bite

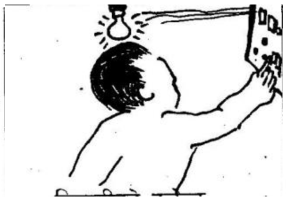

Electric shock

Other specify

6. What was the intent behind the injury event? (Please tick one)

Unintentional ☐ Suicide ☐ Intentional ☐ Unknown ☐

7. What was the outcome of injury? (Please tick as suitable)

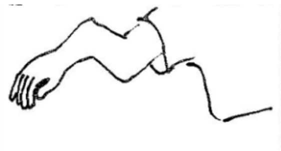

Fracture

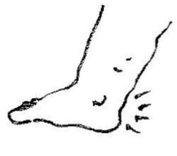

Sprain

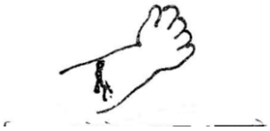

Open wound

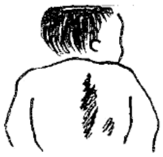

Bruise

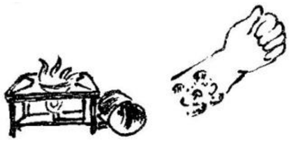

Burn/scald

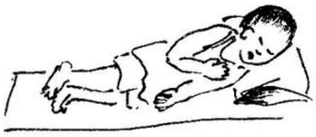

Internal injury

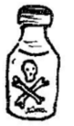

Poisoned

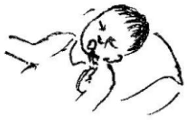

Other specify

8. What was done to the child immediately after the injury?

First Aid only ☐ Hospital Treatment ☐ Not treated ☐ Died ☐

Please mention the place, if treated ☐

Whether the child required to be hospitalised ☐ Yes ☐ No

How much was the costs of treatments? ☐ Medical ☐ Others

9. Please explain how the injury occurred to the child?

☐
